# Bone injury imaging in knee and ankle joints using fast-field-echo resembling a CT using restricted echo-spacing MRI: a feasibility study

**DOI:** 10.3389/fendo.2024.1421876

**Published:** 2024-07-11

**Authors:** Nan Wang, Zhengshi Jin, Funing Liu, Lihua Chen, Ying Zhao, Liangjie Lin, Ailian Liu, Qingwei Song

**Affiliations:** ^1^ Department of Radiology, the First Affiliated Hospital of Dalian Medical University, Dalian, China; ^2^ Clinical and Technical Support, Philips Healthcare, Beijing, China

**Keywords:** MRI, CT, bone, knee, ankle

## Abstract

**Purpose:**

To explore the consistency of FRACTURE (Fast-field-echo Resembling A CT Using Restricted Echo-spacing) MRI and X-Ray/computerized tomography (CT) in the evaluation of bone injuries in knee and ankle joints.

**Methods:**

From Nov. 2020 to Jul. 2023, 42 patients with knee joint or ankle joint injuries who underwent FRACTURE MRI examinations were retrospectively collected. 11 patients were examined by both X-Ray and FRACTURE examinations. 31 patients were examined by both CT and FRACTURE examinations. The fracture, osteophyte, and bone destruction of the joints were evaluated by two radiologists using X-Ray/CT and FRACTURE images, respectively. Kappa test was used for consistency analysis.

**Results:**

The evaluation consistency of fracture, osteophyte and bone destruction via X-Ray and FRACTURE images by radiologist 1 were 0.879, 0.867 and 0.847 respectively, and for radiologist 2 were 0.899, 0.930, and 0.879, respectively. The evaluation consistency of fracture, osteophyte and bone destruction via CT and FRACTURE images by radiologist 1 were 0.938, 0.937 and 0.868 respectively, and for radiologist 2 were 0.961, 0.930, and 0.818, respectively.

**Conclusion:**

For fracture, osteophyte, and bone destruction of knee and ankle joints. FRACTURE MRI showed a high consistency with X-Ray/CT examinations.

## 1 Introduction

Fracture, osteophyte and bone destruction are common types of injuries in bone and joint system, which seriously affect the quality of life of patients. X-Ray and computerized tomography (CT) are the main imaging tools for clinical diagnosis of bone and joint diseases, and widely used in clinical practice. However, X-Ray/CT imaging is associated with ionizing radiation and may cause indeterminate damage to the human body, which requires a high degree of caution for examination of specific populations, such as pediatric patients and pregnant women. Therefore, a non-radiation imaging technology is urgently needed as a supplement to X-Ray or CT examinations especially for multiple longitudinal examinations of disease progress, which can avoid radiation dose to patients, and alleviate the psychological burden of patients and their families (1, 2). MRI, as a non-invasive and non-radiation imaging technique, can provide high soft-tissue contrast with multiple parameters and is considered to be the first choice and necessary examination method for diagnosing soft tissue injuries such as ligaments, tendons and menisci of the osteoarticular system. However, most of the bone structures show very limited signals on conventional MRI (3, 4). A variety of advanced MRI methods are gradually developed for application to the bone and joint system (5). Among them, a modified three dimensional (3D) fast-field-echo sequence termed as Fast-field-echo Resembling A CT Using Restricted Echo-spacing (FRACTURE) can display the bone cortex and show bone changes caused by fracture, osteophyte or bone tumor similar to CT (6, 7). In recent years, applications of FRACTURE MRI in different clinical situations had been reported. FRACTURE MRI can be delineated inwardly and outwardly beveled fractures as well as radiating fracture lines, while standard T1- and T2-weighted MRI detected gunshot-related soft tissue injuries. The FRACTURE MRI was preliminarily explored by Johnson B et al. for children examinations, including evaluations of knee joint, lumbar vertebra and elbow joint (all with only 1 case) (8). Deininger-Czermak E et al. found that visualization of non-pathological bones and fractures at the skull vault on FRACTURE images were comparable to CT images (9). A recent study showed that, with CT as reference, FRACTURE added to standard MR scans, providing a unique contrast of bones from the surrounding tissues, can deliver comparable information with CT on osseous cervical spine status (10).

Actually, several types of MRI techniques can be used for bone visualization. Firstly, the ultrashort echo time (UTE) type methods use a short excitation radio frequency (RF) pulse and the data acquisition is implemented immediately after signal excitation with the start of readout gradient. The 3D UTE imaging is commonly achieved by combining a short rectangular RF pulse excitation with a 3D radial mapping of k-space (11, 12). Zero Echo Time (ZTE) imaging is an alternative approach for imaging of tissues with short transverse relaxation with application of the readout gradient prior to excitation (13, 14). ZTE is less sensitive to gradient imperfections, and can achieve shorter TRs and scan times than UTE due to the minimal gradient switching requirements (15, 16). Different from UTE/ZTE, FRACTURE does not require the TE to be extremely short (such as <1 ms), but uses a multi-echo 3D fast field echo sequence for signal acquisition at fixed echo intervals. And the high contrast bone images with high SNR can be reconstructed through echo accumulation, echo subtraction, and grayscale inversion (17, 18). FRACTURE places much lower requirements on hardware performance such as on gradient or RF pulse.

While the application of FRACTURE in evaluation of knee and ankle joint injuries still have not been comprehensively reported. The study objective was to assess whether the 3D isotropic FRACTURE imaging is feasible in evaluation of knee and ankle joint injuries with X-Ray/CT imaging as reference.

## 2 Methods

### 2.1 Subjects

**Figure 1:**
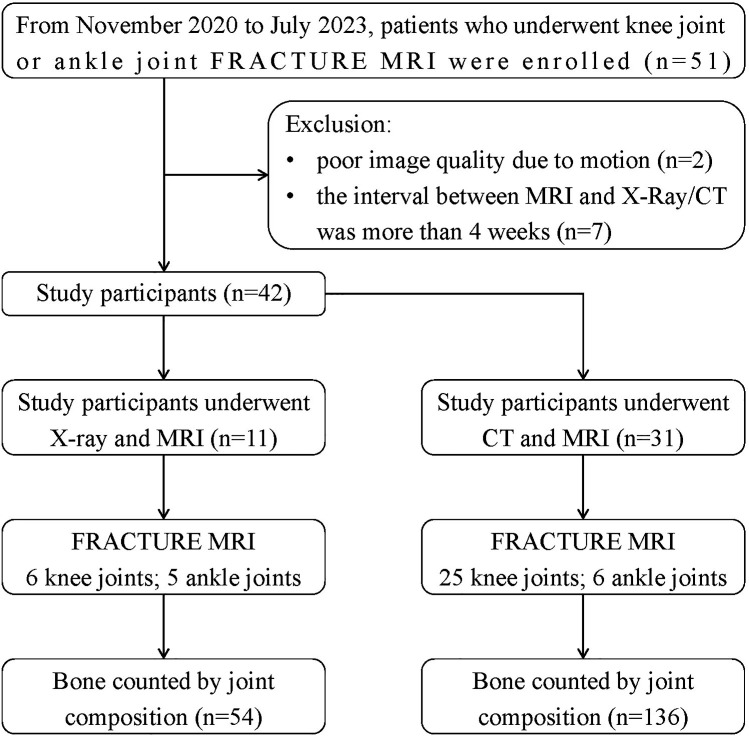
Flowchart of participants that were included and evaluated in the study.

From Nov. 2020 to Jul. 2023, 42 patients with knee joint or ankle joint who underwent FRACTURE MRI examinations in our hospital were retrospectively collected. Inclusive criteria: (1) complete MRI (including FRACTURE) and X-Ray or CT scans of the same examination issue; (2) detailed clinical history. Exclusion criteria: (1) poor image quality due to motion; (2) the interval between MRI and X-Ray/CT was more than 4 weeks. 11 patients were examined by both X-Ray and FRACTURE examinations, including 7 males and 4 females, aged from 25 to 78 years, including 6 knee joints (4 left and 2 right) and 5 ankle joints (3 left and 2 right). 31 patients were examined by both CT and FRACTURE examinations, including 18 males and 13 females, aged from 16 to 82 years, including 25 knee joints (13 left and 12 right) and 6 ankle joints (3 left and 3 right). The flowchart can be seen in **Figure 1**.

This study was approved by the Institutional Review Board of our hospital (PJ-KS-XJS-2022-64). The requirement for the informed consent was waived due to the retrospective nature of the study.

### 2.2 Image acquisition

MRI scans were performed on a 3.0T MRI scanner (Ingenia CX, Philips Healthcare, Best, the Netherlands). The knee joint was scanned with a 16-channel knee coil, and the ankle joint was scanned with an 8-channel foot coil. The FRACTURE imaging was scanned in the sagittal orientation for both knee and ankle joints. Detailed imaging parameters are listed in **Table 1**. The FRACTURE MRI was acquired with six gradient echoes using in-phase echo-spacing (first TE: 2.3 ms; echo-spacing: 2.3 ms). Echo times at in-phase intervals enabled accentuated T2 decay of bones, while the signals from other tissues were preserved. As the signal decreased from the first to the sixth echoes, the contrast increased. The summation of all six echoes yielded cumulated images with high contrast by maintaining a high signal to noise ratio (SNR). Dephasing across voxel was additionally minimized due to the high scan resolution. The cumulated images were finally inverted so that they resembled the CT images. Echo summation and inversion of the cumulated images were automatically performed on the host computer, and the reconstructed FRACTURE images were translated to the IntelliSpace Portal (ISP v9.0, Philips Healthcare) for further analyses.

**Table 1:** Parameter settings of FRACTURE MRI.

	FRACTURE (knee)	FRACTURE (ankle)
Field-of-view (mm3)	160×160×105	160×160×102
Acquisition voxel size (mm2)	0.6×0.6	0.6×0.6
Matrix size	268×264	268×264
Slice/Thick (mm)	350/0.6	340/0.6
TR (ms)	18	18
First TE (ms)	2.3	2.3
Echo-space (ms)	2.3	2.3
Echo number	6	6
Flip angle (°)	15	15
Compressed SENSE factor	CS 6	CS 6
Scan time (min: sec)	03: 07	02: 58

FRACTURE, FFE Resembling A CT Using Restricted Echo-spacing; TR, repetition time; TE, echo time.

CT examination was performed on the Discovery HD 750 CT scanner (GE Healthcare, USA). The tube voltage was 100kV, the tube current was 150mA, and the adaptive statistical iterative reconstruction (ASIR) was 30%. The CT scan range covered the whole knee joint or ankle joint. After data scan, the image was automatically transmitted to GE workstation (AW4.6) for analysis.

X-Ray examination was performed on the beam limiting device (General Medical Merate, Italy). X-Ray images of knee and ankle joints included both positive and lateral positions. After the filming was completed, the images were automatically transmitted to a picture archiving and communication system (PACS) workstation certifed for clinical use (miPlatform, Client ID: 3.0.30501.283).

### 2.3 Image analysis

Coronal and sagittal CT and FRACTURE images were further reconstructed by a technician with 4 years’ work experience, and the reconstructed slice thickness and interval were both 1 mm. Fracture is defined as continuous interruption of cortical bone. Osteophyte is a fibrocartilage-capped bony outgrowth. Bone destruction is defined as loss of bone tissue caused by local bone being replaced by diseased tissue. Two radiologists with five and seven years’ experience respectively in imaging diagnosis independently evaluated the FRACTURE and X-Ray/CT images to determine whether each piece of bone had the following three signs: fracture, osteophyte, and bone destruction by a blind method. The images were repeatedly read by the two radiologists with a time interval more than 4 weeks in a random order on a PACS workstation certified for clinical use.

### 2.4 Statistical analysis

Data organization and statistical analyses were performed using SPSS version 22.0 software. Kappa test was used for consistency analyses, which includes consistency between results evaluated by FRACTURE and X-Ray/CT images for each radiologist, consistency between results by the two radiologists using different imaging techniques,.Kappa’s criteria for checking consistency are as follows: 0-0.2 for extremely low consistency, 0.2-0.4 for general consistency, 0.4-0.6 for medium consistency, 0.6-0.8 for high consistency, and 0.8-1 for almost complete consistency.

## 3 Results

### 3.1 Bone compositions for the knee and ankle joints

The knee and ankle joints of all patients were counted according to the bone compositions. As for the 11 patients who underwent X-Ray and FRACTURE examinations, totally 54 pieces of bones were analyzed, including 6 pieces of femur and patella, 11 pieces of tibia and fibula, and 5 pieces of scaphoid, medial cuneiform, lateral cuneiform and calcaneus, respectively. As for the 31 patients who underwent CT and FRACTURE examinations, totally 136 pieces of bones were analyzed, including 25 pieces of femur and patella, 31 pieces of tibia and fibula, 6 pieces of scaphoid, medial cuneiform, lateral cuneiform and calcaneus, respectively. Results of lesion evaluation on all these bones by two radiologists were listed in **Tables 2** and **3**. Radiologist 1 diagnosed 35 bones of fracture, 37 bones of osteophyte and 15 bones of bone destruction through X-Ray and CT, and 34 bones of fracture, 42 bones of osteophyte and 17 bones of bone destruction through FRACTURE. Radiologist 2 diagnosed 38 bones of fracture, 34 bones of osteophyte and 19 bones of bone destruction through X-Ray and CT, and 41 bones of fracture, 36 bones of osteophyte and 14 bones of bone destruction through FRACTURE.

**Table 2:** Total number of different kinds of bone lesions by X-Ray and FRACTURE images (n=54).

	Radiologist 1		Radiologist 2	
	X-Ray	MRI	X-Ray	MRI
fracture	5	4	5	6
non-fracture	49	50	49	48
osteophyte	8	10	9	8
non-osteophyte	46	44	45	46
bone destruction	4	3	5	4
non-bone destruction	50	51	49	50

**Table 3:** Total number of different kinds of bone lesions by CT and FRACTURE images (n=136).

	Radiologist 1		Radiologist 2	
	CT	MRI	CT	MRI
fracture	33	30	33	35
non-fracture	103	106	103	101
osteophyte	29	32	25	28
non-osteophyte	107	104	111	108
bone destruction	11	14	14	10
non-bone destruction	125	122	122	126

### 3.2 Consistency of assessment of lesions

**Figure 2:**
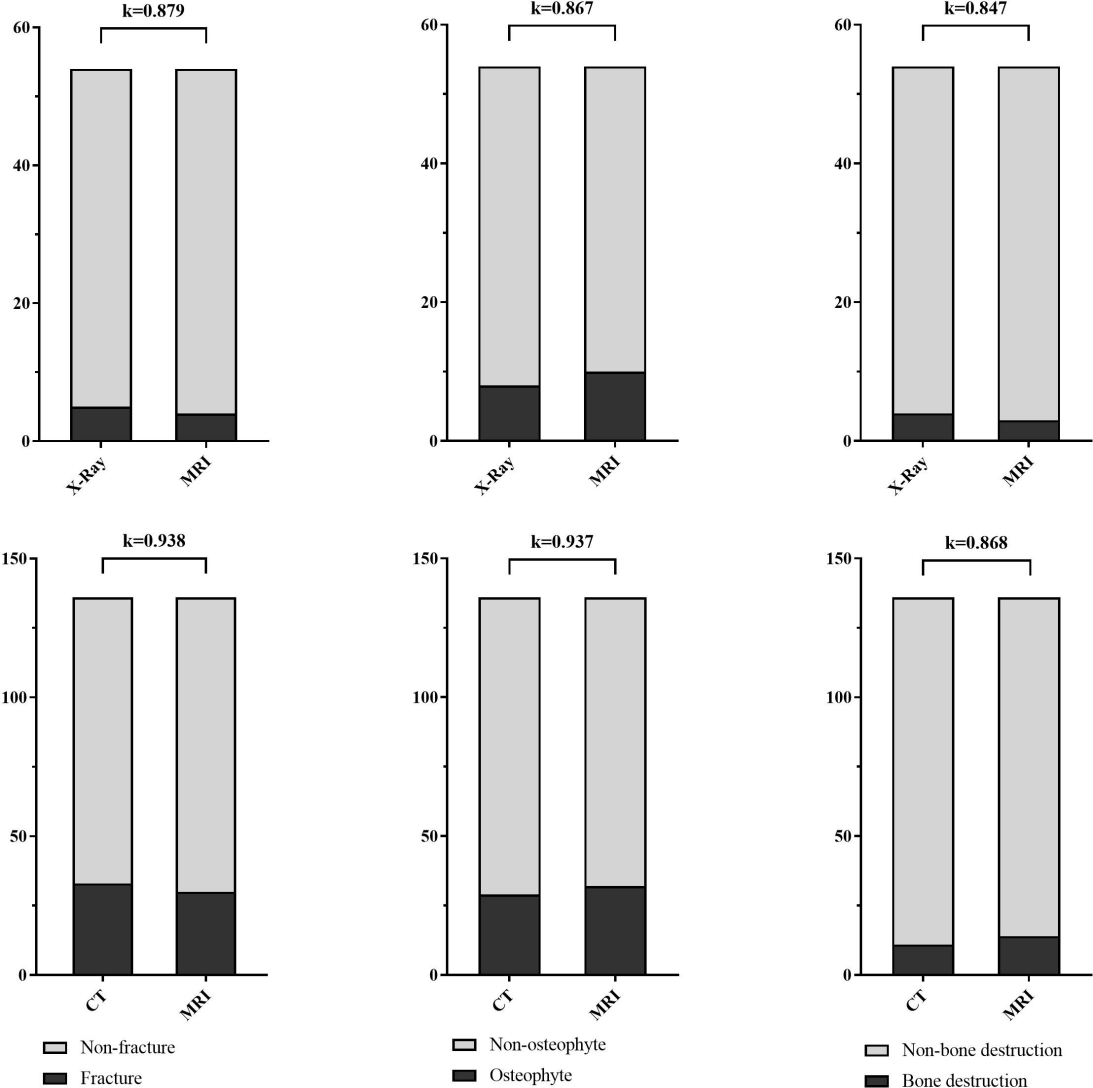
Stacked bar charts show the fracture, osteophyte and bone destruction assessed by radiologist 1 via X-Ray/CT and FRACTURE images were with high consistency (kappa range: 0.847-0.938).

**Figure 3:**
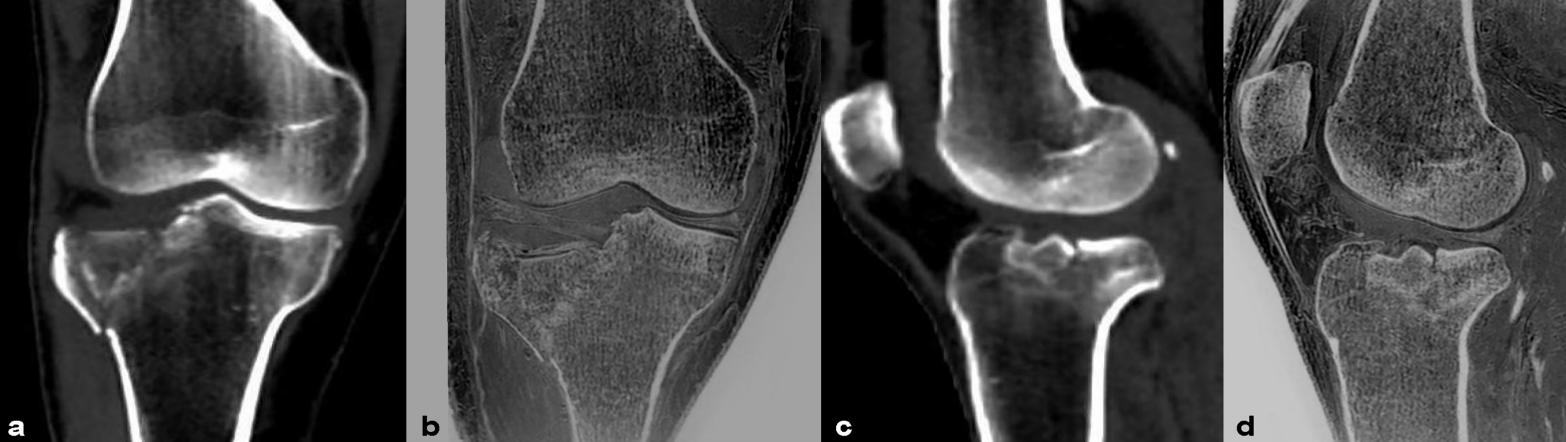
Images in a 45-year-old male with fracture of the right tibial plateau. **(A, B)** were coronal CT and FRACTURE images. **(C, D)** were sagittal CT and FRACTURE images. CT and FRACTURE images showed the same oblique fracture line and bone fragment displacement.

**Figure 4:**
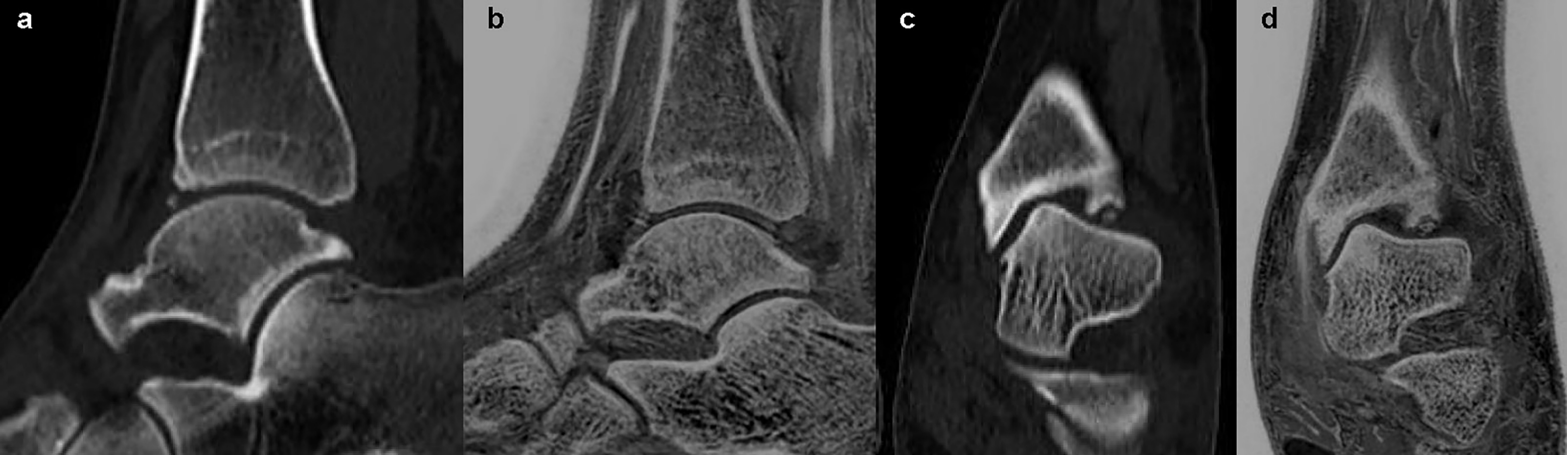
Images in a 36-year-old female with anterior and inferior avulsion fracture of left tibia. **(A, C)** were sagittal and coronal CT images. **(B, D)** were sagittal and coronal FRACTURE images. Free bone fragments were consistent in CT and FRACTURE images.

**Figure 5:**
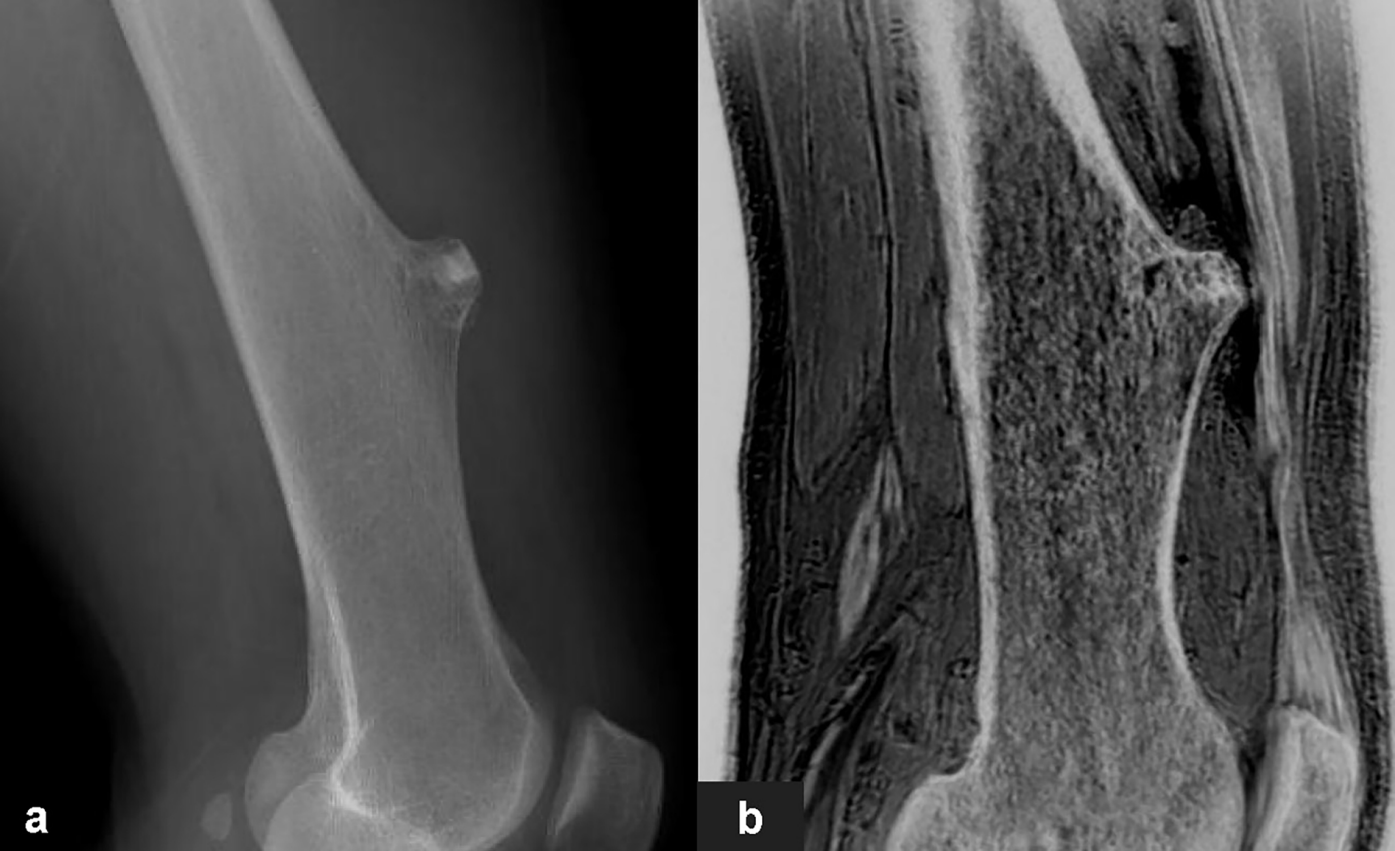
Images in a 65-year-old male with osteochondroma of the left lower femoral shaft. **(A, B)** were lateral X-Ray and sagittal FRACTURE images. Wide base and morphology of tumor were consistent in X-Ray and FRACTURE images.

**Figure 6:**
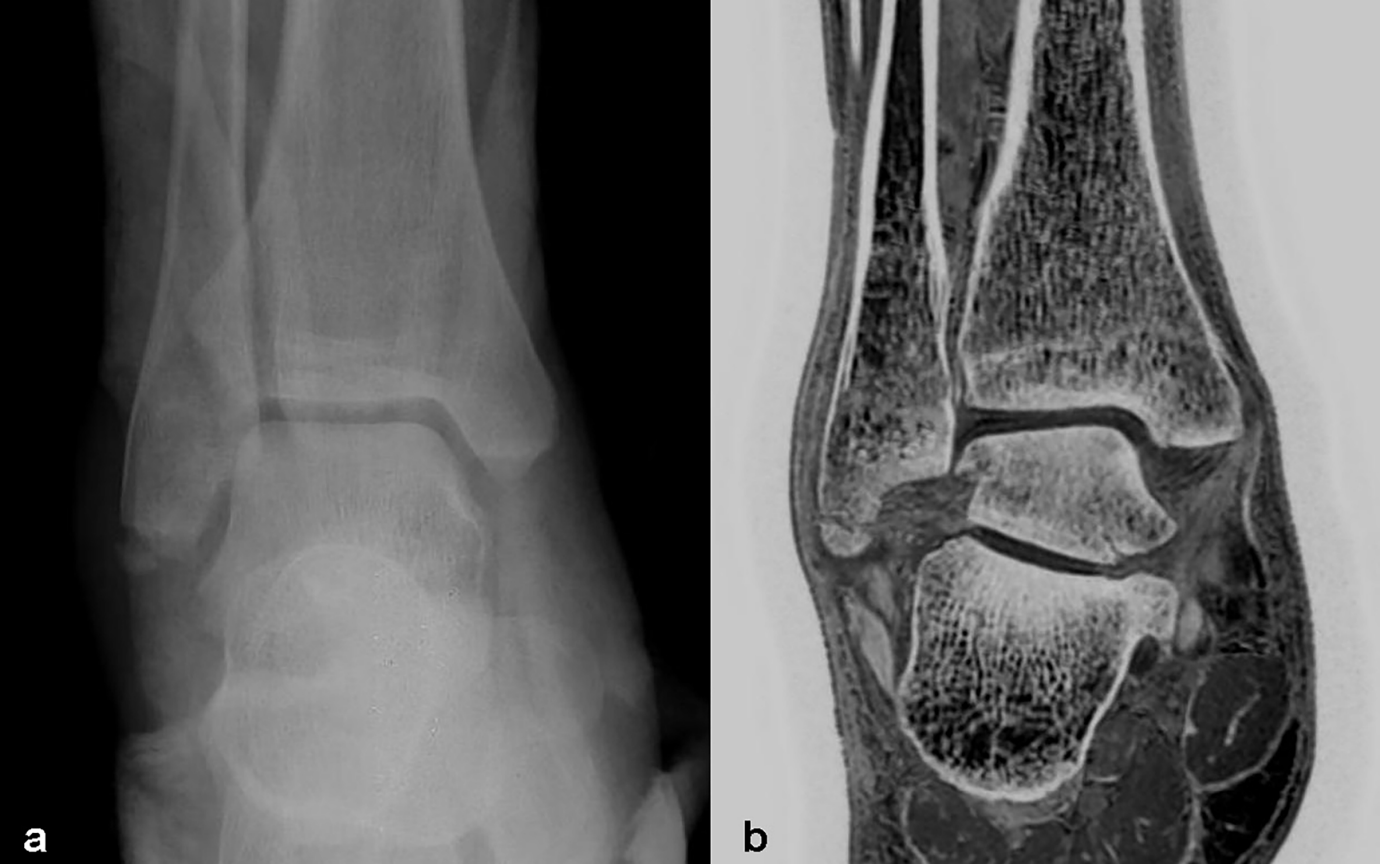
Images in a 40-year-old male with right distal fibula fracture. **(A, B)** were positive X-Ray and coronal FRACTURE images. Fracture lines and bone fragments were consistent in X-Ray and FRACTURE images.

The fracture, osteophyte and bone destruction assessed by radiologist 1 via X-Ray and FRACTURE images were all with high consistency (kappa values of 0.879, 0.867 and 0.847 respectively), and similar results were obtained for radiologist 2 (kappa values of 0.899, 0.930, and 0.879). The fracture, osteophyte and bone destruction assessed by two radiologists via CT and FRACTURE images were also with high consistency (kappa values of 0.938, 0.937 and 0.868 for radiologist 1, and kappa values of 0.961, 0.930, and 0.818 for radiologist 2). The fracture, osteophyte and bone destruction assessed by two radiologists via X-Ray/CT were with high consistency (kappa range: 0.868-1). The fracture, osteophyte and bone destruction assessed by two radiologists via FRACTURE were with high consistency (kappa range: 0.780-0.915) (**Figures 2**–**6**; **Tables 4**, **5**).

**Table 4:** Consistency of lesion evaluation by the two radiologists using X-Ray and FRACTURE MRI.

	Radiologist 1	Radiologist 2	MRI	X-Ray
	X-Ray vs. MRI	X-Ray vs. MRI	Radiologist 1 vs. 2	Radiologist 1 vs. 2
fracture	0.879	0.899	0.780	1
osteophyte	0.867	0.930	0.867	0.930
bone destruction	0.847	0.879	0.847	0.879

**Table 5:** Consistency of lesion evaluation by the two radiologists using CT and FRACTURE MRI.

	Radiologist 1	Radiologist 2	MRI	CT
	CT vs. MRI	CT vs. MRI	Radiologist 1 vs. 2	Radiologist 1 vs. 2
fracture	0.938	0.961	0.899	1
osteophyte	0.937	0.930	0.915	0.908
bone destruction	0.868	0.818	0.818	0.868

## 4 Discussion

In our study, evaluations of bone lesions on FRACTURE and X-Ray/CT images were in high consistency (kappa range: 0.818-0.961), especially for the evaluation of fracture and the display of fracture line, which indicates the reliable performance of FRACTURE. Besides, evaluations of osteophyte and bone destruction of knee or ankle joints on FRACTURE images by different radiologists showed high consistency, which indicates the high stability and repeatability of FRACTURE imaging. FRACTURE combined with conventional MRI enables simultaneous visualization of both bone and soft tissue injuries, which would be of great potential for direct and comprehensive assessment of diseases in the osteoarticular system.

There was no study on the consistency analyses between X-Ray/CT and UTE on the evaluation of bone lesions in the lower extremities. Recently, some scholars had compare the diagnostic performance of CT-like images based on a 3D T1 GRE, UTE, and FRACTURE with conventional CT in patients with suspected osseous shoulder pathologies (19). Assessment of bony Bankart lesions and other osseous pathologies was feasible and accurate using CT-like images based on 3-T MRI compared with conventional CT. Compared to the T1 GRE and FRACTURE sequence, the UTE measurements correlated best with CT. The bone tumors or tumor-like lesions in the lower extremities were analyzed in previous literature reports (20), where the consistency in visualization of periosteal reaction and penetration of the cortex was fair to good to excellent between ZTE and CT (kappa range: 0.682–0.852). In a single-center data, the result of osteoarthritis scoring of CT and sCT images set shows that sCT provides comparable scoring accuracy to CT for knee osteoarthritis scoring. The intermodality agreement of osteoarthritis scores between CT and sCT was substantial to almost-perfect for tibiofemoral (k = 0.63 and 0.84 for the two readers) and patellofemoral joints (k = 0.78 and 0.81 for the two readers), where both are showing acceptable confidence levels for scoring osteoarthritis (21).

FRACTURE, UTE and ZTE have different methods to highlight the bone structure. FRACTURE images are post-processed (signal inversion) to improve bone contrast. UTE/ZTE images can also be post-processed to improve bone contrast (22). UTE/ZTE images can be combined with long T2 suppression pulses to create high bone contrast (23, 24). Deininger-Czermak et al. compared UTE and FRACTURE for the assessment of craniocervical junction in subjects with varying degrees of degeneration using CT imaging as reference, and concluded that UTE and FRACTURE can both be used as valid alternatives to CT in evaluation of the craniocervical junction in nontraumatic cases (25). In addition to direct scanning to highlight the bony tissues. MR-based synthetic CT (sCT) is an alternative solution for generating quantitative CT-like contrast from MR images. SCT was generated using a patch-based neural network derived from a UNet (26). Some neural network models have been developed into bone MRI software. With CT as the reference standard, synthetic CT of the sacroiliac joints has better diagnostic performance in the detection of structural lesions in individuals suspected of having sacroiliitis compared with routine T1-weighted MRI (27). The advantage of sCT is that there is no false positive bone visualizations at the water-fat interfaces (28). However, the neural network reconstruction image does not mean that it is the real performance. Different studies applied different deep learning models (29). It still needs a lot of multi-center verification. Moreover, the T1 GRE sequence required by neural network is not a routine clinical diagnostic sequence, and it also needs additional scanning. Thus, Bone-MRI software is rarely used in clinic, and limited to the research stage.

We evaluated the diagnostic value of MR derived CT-like images based on the FRACTURE sequence for assessment of fracture, osteophyte and bone destruction in knee and ankle joints, and the results were compared with references, which consisted of conventional X-Ray/CT findings. Bone injuries assessed by FRACTURE imaging showed a high consistency with findings by X-Ray/CT imaging. Therefore, FRACTURE can be used as a reliable alternative of X-Ray/CT examination for fracture, osteophyte and bone destruction in knee and ankle joints especially for patients who need to avoid of ionizing radiation, including infants and pregnant women.

Besides, FRACTURE is associated with the following three advantages for clinical applications: First, it is based on conventional 3D gradient echo sequences available on most commercially available MRI scanners; Second, 3D acquisition ensures the image resolution and SNR, and the isotropic imaging facilitates multiplanar reconstruction for visualization of the joints or lesions in any direction; and third multi-echo acquisition at fixed intervals to minimize the chemical shift artifact and ensure the clarity of final images. The scan time may be an issue of FRACTURE. While, with introduction of the compressed sensing technology for acquisition acceleration (30), the FRACTURE imaging with spatial resolution similar to CT scans can be completed in a significantly reduced scan time of about 3 mins (previously around 6 mins) without degeneration in image quality (31, 32).

Fracture, osteophyte, and bone destruction are common diseases of bone and joint system,having typical imaging manifestations and FRACTURE MRI should not only be valid for evaluation of these kinds of lesions in knee and ankle joints, but also applicable to regions such as spine for evaluation of osteophyte and compression fracture etc., especially for patients with spinal canal stenosis caused by osteopathy. MRI examination that takes into account both spinal bone changes and intervertebral disc lesions is worthy of further study.

## 5 Limitations and future work

Our research has some limitations. First, FRACTURE sequence causes all tendons, ligaments, air pockets, water/fat interfaces to end up as high intensity false positives. Undeniably, false positive bone visualisations limit that single application of the FRACTURE sequence. However, whether it is trauma or bone tumor patients, conventional MRI sequences including T1, T2 and PDWI are indispensable. Conventional MRI is used to display soft tissues including tendons, ligaments, etc. FRACTURE is a supplement to conventional MRI examination, showing bone. False positive bone visualizations will not lead to wrong diagnosis in clinical work. Second, another concern of FRACTURE is its high sensitivity to the strong susceptibility at the bone and soft tissue interface (especially for the trabecular bone and marrow interface in the spine/hip). Other T2* shortening factors, such as iron deposition (e.g., iron in the spine, which might significantly shorten its T2*), multiple peaks for marrow fat (thus short T2* for marrow, especially at high field strength such as 7T), and field inhomogeneity, might contribute to the overestimation of bone in FRACTURE imaging. Third, the sample size was relatively small, and assessment of FRACTURE for more types of bone and joint diseases, such as osteochondroma and giant cell tumor, were not available, which need to be further investigated. Fourth, CT and X-Ray images were not both acquired for the same patients, which may introduce bias in the performance evaluation of FRACTURE.

In our further studies, the FRACTURE method will be assessed on a larger cohort of patients (particularly for patient cohorts who need to avoid of ionizing radiation), and more types of bone injuries will be included for evaluation.

## 6 Conclusion

FRACTURE is an easily accessible and implementable MRI method for bone injury examination. The addition of FRACTURE to conventional MRI provides the opportunity to simultaneously obtain osseous and soft tissue injury information within the same examination, and results from our study suggest that FRACTURE enables clear assessments of three types of bone lesions on knee and ankle joints: fracture, osteophyte and bone destruction, which were in high consistency with results by X-Ray/CT examinations. In the future, it is a valuable research direction for people who need to reduce or avoid electron radiation.

## Data availability statement

The datasets utilized in the present study can be obtained from the corresponding author upon reasonable request.

## Ethics statement

The studies involving humans were approved by the Institutional Review Board, First Affiliated Hospital of Dalian Medical University (PJ-KS-XJS-2022-64). The studies were conducted in accordance with the local legislation and institutional requirements. Written informed consent for participation was not required from the participants or the participants’ legal guardians/next of kin in accordance with the national legislation and institutional requirements.

## Author contributions

NW: Writing – original draft, Data curation, Formal analysis, Methodology. ZJ: Data curation, Writing – original draft, Project administration. FL: Data curation, Writing – original draft, Methodology. LC: Methodology, Writing – original draft, Validation. YZ: Data curation, Software, Writing – original draft, Investigation. LL: Software, Writing – original draft. AL: Writing – review & editing, Software, Supervision, Validation. QS: Writing – review & editing, Conceptualization, Project administration, Software, Supervision.
